# Point-of-care ultrasound detection of tracheal wall thickening caused by smoke inhalation

**DOI:** 10.1186/2036-7902-6-11

**Published:** 2014-07-09

**Authors:** Toru Kameda, Masato Fujita

**Affiliations:** 1 Department of Emergency Medicine, Red Cross Society Azumino Hospital, 5685 Toyoshina, Azumino, Nagano 399-8292, Japan; 2 Department of Emergency and Intensive Care Medicine, Red Cross Society Azumino Hospital, 5685 Toyoshina, Azumino, Nagano 399-8292, Japan

**Keywords:** Ultrasonography, Point-of-care, Trachea, Smoke inhalation

## Abstract

Smoke inhalation is the leading cause of death due to fires. When a patient presents with smoke inhalation, prompt assessment of the airway and breathing is necessary. Point-of-care ultrasonography (US) is used for the rapid assessment of critically ill or injured patients. We herein present a case report of a 54-year-old male who was transferred to the emergency department with shortness of breath, coughing, carbonaceous sputa, and rhinorrhea after inhaling smoke caused by a fire in his locked bedroom. He had no surface burns on the face and no edema or erosion in the oral cavity. He had hoarseness without stridor. His breath sounds were positive for expiratory wheezes. Laryngoscopy showed light edema and erosive findings on the supraglottic region. Bedside point-of-care US revealed hypoechoic thickening of the tracheal wall. The thickening was confirmed by a computed tomographic scan. The patient was carefully monitored with preparation for emergency airway management and was treated with supplemental oxygen and an aerosolized beta-2 adrenergic agonist in the intensive care unit. The symptoms were subsequently relieved, and reexamination by US after 2 days showed remission of the wall thickening. Point-of-care US may therefore be a useful modality for the rapid diagnosis and effective follow-up of tracheal wall thickening caused by smoke inhalation.

## Background

Smoke inhalation is the leading cause of death due to fires [1]. When a patient presents with smoke inhalation, prompt assessment of the airway and breathing is necessary. Point-of-care ultrasonography (US) is used for the rapid diagnostic assessment and the procedural guidance of critically ill or injured patients [2]. It is easily repeatable; therefore, it is an ideal imaging modality for close observation of such patients [2]. Point-of-care US is now being used for airway assessment and management in the emergency, critical care, and anesthetic settings [3,4]. However, to the best of our knowledge, the use of US for the detection of tracheal wall thickening caused by smoke inhalation has never been reported in the English literature. We herein present a case report of a patient presenting with smoke inhalation whose tracheal wall thickness was evaluated repeatedly with point-of-care US.

## Case presentation

A 54-year-old male was transferred to the emergency department with shortness of breath, coughing, carbonaceous sputa, and rhinorrhea after inhaling smoke caused by a fire. Approximately 6 h before arrival, he was caught in a fire which started on the ground floor of his house while he was sleeping upstairs in a locked bedroom. He inhaled considerable smoke without direct exposure to the flames. When he was rescued from the site, he was unaware of these symptoms. The symptoms became increasingly evident. He had a past medical history of ischemic stroke without long-term neurological sequelae.

**Figure 1:**
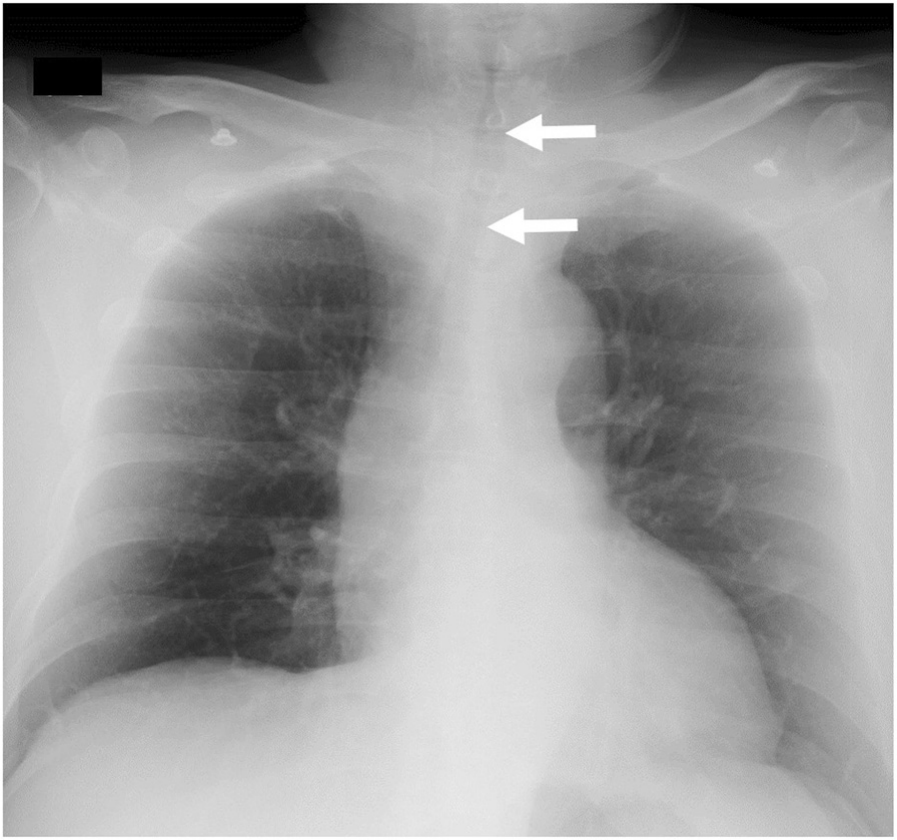
**A chest X-ray taken on admission.** The chest X-ray image indicated narrowing of the trachea (arrows).

**Figure 2:**
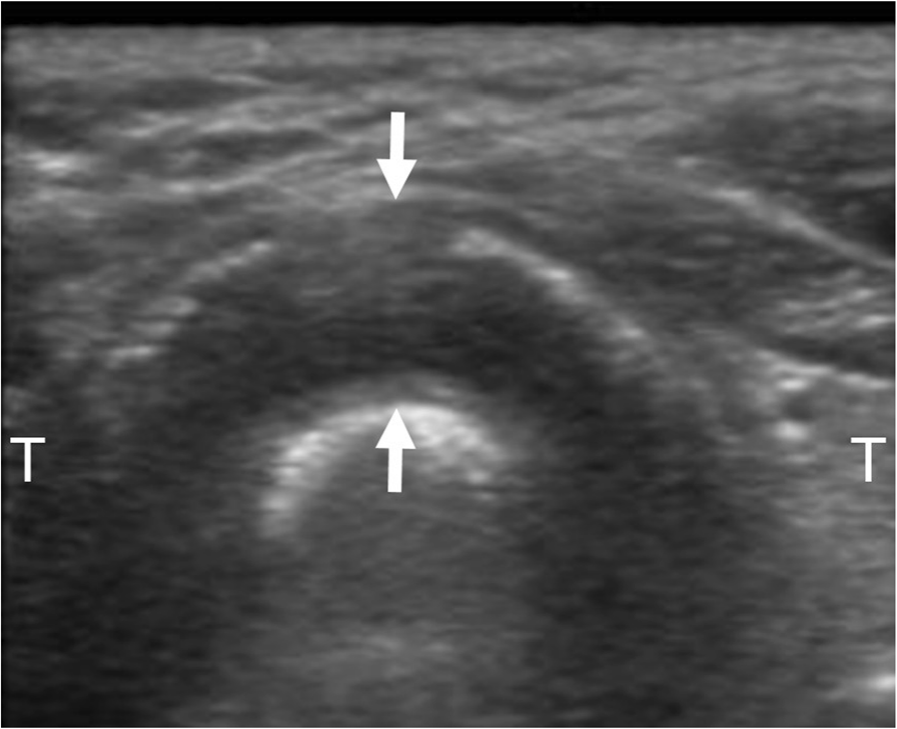
**The transverse view of the tracheal US on admission.** The US image showed hypoechoic thickening of the tracheal wall at the level cranially adjacent to the thyroid isthmus (arrows). T indicates the thyroid gland.

**Figure 3:**
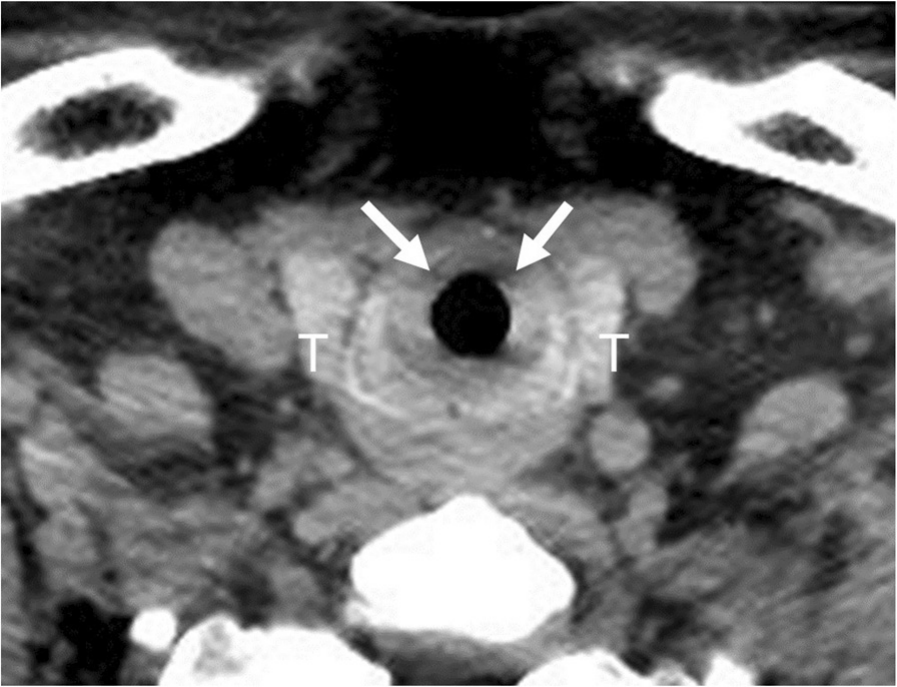
**The CT scan performed on admission.** The CT image showed thickening of the tracheal wall (arrows). T indicates the thyroid gland.

On examination, the patient was alert. His oxygen saturation was 94% on 2 L of oxygen by nasal cannula, with a respiratory rate of 25 breaths/min. His heart rate was 106 beats/min, his blood pressure was 151/100 mmHg, and his body temperature was 37.3°C. He had no surface burns on the face and no edema or erosions in the oral cavity. He had hoarseness without stridor. His breath sounds were positive for expiratory wheezes. His carboxyhemoglobin concentration was 3.3% on admission.Chest X-rays indicated narrowing of the trachea (Figure 1). Fiberoptic laryngoscopy showed light edema and erosive findings in the supraglottic region, while white-colored edematous findings were visible through the vocal cords in the infraglottic region. Bedside point-of-care US with a 6- to 13-MHz linear probe (MicroMaxx; SonoSite, Bothell, WA, USA) revealed hypoechoic thickening of the anterior part of the tracheal wall. The section at the level cranially adjacent to the thyroid isthmus appeared to be thickest in the anterior portion detectable with US. The thickness from the outer edge of the hypoechoic tracheal ring to the outer edge of the hyperechoic air-mucosa interface was 9.0 mm (Figure 2). The thickening was subsequently confirmed by a non-enhanced computed tomography (CT) scan (Figure 3), in which no pulmonary parenchymal injury was observed.

**Figure 4:**
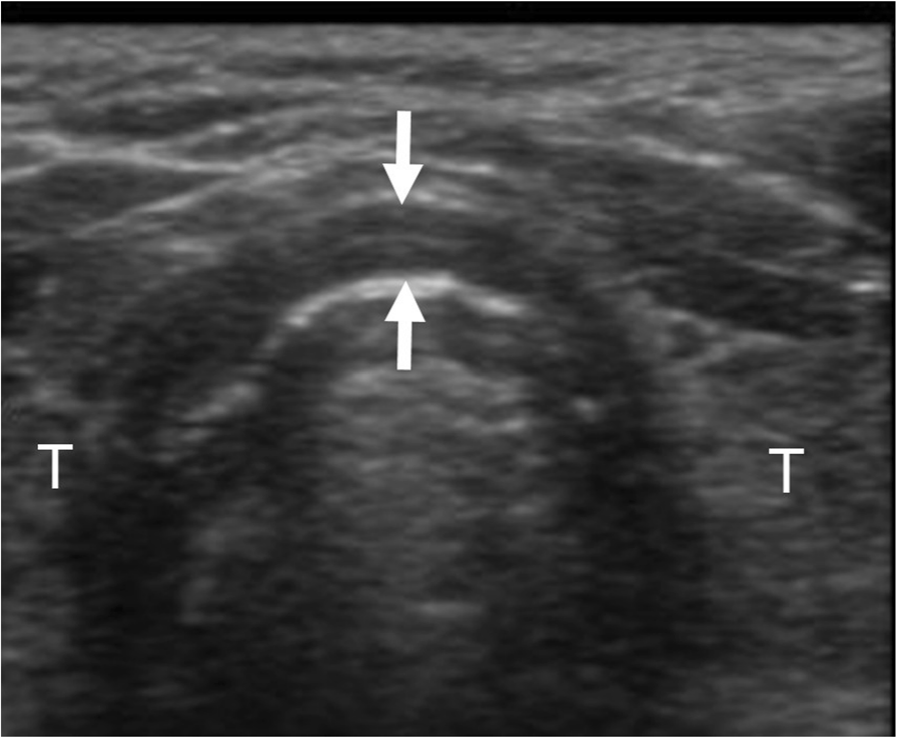
**A transverse view of tracheal US obtained at the same level 2 days after admission.** The US image showed remission of the tracheal wall thickening (arrows). T indicates the thyroid gland.

The patient was carefully monitored with preparation for emergency airway management and was treated with supplemental oxygen and an aerosolized beta-2 adrenergic agonist in the intensive care unit (ICU). The symptoms were subsequently relieved, and reexamination by point-of-care US after 2 days showed remission of the wall thickening. The thickness at the same level was 2.9 mm (Figure 4). Resolution of the supra- and infraglottic lesions was also confirmed by follow-up laryngoscopy 8 days after admission, and the patient was discharged from the hospital 12 days after the accident.

## Discussion

Smoke inhalation injury should be strongly suspected in the presence of a specific history and physical examination [5,6] and is commonly confirmed by endoscopic examination [5-8]. Fiberoptic bronchoscopy is considered the gold standard for the visualization and evaluation of smoke inhalation injury [6]. However, it may not be readily available around the clock in some institutions. Moreover, it may be uncomfortable for some patients and requires careful anesthesia to avoid airway issues. On the other hand, fiberoptic laryngoscopy is better tolerated by patients and is often more readily available than bronchoscopy in emergency settings. Muehlberger et al. described the efficacy of fiberoptic laryngoscopy for predicting the airway integrity in patients with a possibility of less severe smoke inhalation injuries [7].

US can allow the identification of tracheal stenosis from various causes [9]. In the present case without facial and neck burns, the anterior wall thickening of the upper trachea was quickly detected with point-of-care US immediately after laryngoscopic evaluation. The tracheal wall consists of the tunica adventitia, tracheal cartilage, and mucosa containing the tracheal glands [10]. In normal volunteers, hypoechoic cartilaginous rings and a hyperechoic air-mucosa interface are detectable on US [3,9,11]. Shih et al. reported anterior tracheal wall thicknesses on US of 1.5 ± 0.2 mm and 1.2 ± 0.2 mm in normal male and female volunteers, respectively. In that study, the thickness was defined as the distance from the inner border of the thyroid isthmus to the outer edge of the hyperechoic air-mucosa interface on a transverse section [9]. We measured the thickness of the anterior wall at the level cranially adjacent to the thyroid isthmus that appeared to be the thickest section. Surprisingly, the tracheal wall thickness reached 9.0 mm on admission. The degree of thickening, which is thought to be primarily induced by mucosal edema [12,13], decreased after 2 days, although it did not normalize. Point-of-care US may be the first modality of choice for the initial evaluation of the upper trachea in patients who suffer smoke inhalation. This noninvasive method may also be useful for repeated evaluation of the proximal airway in conjunction with fiberoptic laryngoscopy in patients with less severe smoke inhalation injuries.

Recently, several papers on laryngeal US have been published on other pathologies [14,15]. Although we did not observe the laryngeal region with US in this case, we think that, based on these papers, laryngeal US may also be useful to detect any additional pathological changes caused by smoke inhalation.

The tracheal wall thickening detected with US was confirmed with a CT scan in this case. Although it has been shown that chest CT scans can precisely detect the extent of complications such as acute respiratory distress syndrome and pneumonia in patients with smoke inhalation [16,17], the detection of the tracheal wall thickening caused by smoke inhalation with a CT scan had also never been reported previously. There was one report on the relationship between the bronchial wall thickness detected with a CT scan to the total number of ventilator days, the ICU stay, and the development of pneumonia [17]. We think that further studies are warranted to determine whether the tracheal wall thickness measured with US or CT scans can be used as an early predictor of severity and complications. However, our patient with significant tracheal wall thickening recovered without any complications and did not require tracheal intubation.

## Conclusions

Point-of-care US may be a useful modality for the rapid diagnosis and follow-up of tracheal wall thickening caused by smoke inhalation.

## Consent

Written informed consent was obtained from the patient for publication of this case report and any accompanying images. A copy of the written consent is available for review by the Editor-in-Chief of this journal.

## Competing interests

The authors declare that they have no competing interests in association with this study.

## Authors' contributions

TK drafted and edited the manuscript. MF edited the manuscript. Both authors read and approved the final manuscript.
